# CONAN: copy number variation analysis software for genome-wide association studies

**DOI:** 10.1186/1471-2105-11-318

**Published:** 2010-06-14

**Authors:** Lukas Forer, Sebastian Schönherr, Hansi Weissensteiner, Florian Haider, Thomas Kluckner, Christian Gieger, Heinz-Erich Wichmann, Günther Specht, Florian Kronenberg, Anita Kloss-Brandstätter

**Affiliations:** 1 Division of Genetic Epidemiology, Department of Medical Genetics, Molecular and Clinical Pharmacology, Innsbruck Medical University, 6020 Innsbruck, Austria; 2 Department of Database and Information Systems, Institute of Computer Science, University of Innsbruck, 6020 Innsbruck, Austria; 3 Institute of Epidemiology, Helmholtz Center Munich, German Research Center for Environmental Health, 85764 Neuherberg, Germany; 4 Institute of Medical Informatics, Biometry and Epidemiology, Chair of Epidemiology, Ludwig-Maximilians-Universität, 80539 Munich, Germany; 5 Klinikum Großhadern, 80337 Munich, Germany

## Abstract

**Background:**

Genome-wide association studies (GWAS) based on single nucleotide polymorphisms (SNPs) revolutionized our perception of the genetic regulation of complex traits and diseases. Copy number variations (CNVs) promise to shed additional light on the genetic basis of monogenic as well as complex diseases and phenotypes. Indeed, the number of detected associations between CNVs and certain phenotypes are constantly increasing. However, while several software packages support the determination of CNVs from SNP chip data, the downstream statistical inference of CNV-phenotype associations is still subject to complicated and inefficient in-house solutions, thus strongly limiting the performance of GWAS based on CNVs.

**Results:**

CONAN is a freely available client-server software solution which provides an intuitive graphical user interface for categorizing, analyzing and associating CNVs with phenotypes. Moreover, CONAN assists the evaluation process by visualizing detected associations via Manhattan plots in order to enable a rapid identification of genome-wide significant CNV regions. Various file formats including the information on CNVs in population samples are supported as input data.

**Conclusions:**

CONAN facilitates the performance of GWAS based on CNVs and the visual analysis of calculated results. CONAN provides a rapid, valid and straightforward software solution to identify genetic variation underlying the 'missing' heritability for complex traits that remains unexplained by recent GWAS. The freely available software can be downloaded at http://genepi-conan.i-med.ac.at.

## Background

Genome-wide association studies (GWAS) have identified associations between various phenotypes and common sequence polymorphisms, which might play a role for disease development (for a comprehensive overview see [1]). For most common diseases, these discoveries collectively explain only a modest fraction (1-15%) of heritable variation of the phenotype [2]. Recently, genome re-sequencing studies demonstrated that most bases that vary among human genomes reside in copy number variations (CNVs) [3]. CNVs are genomic segments which are duplicated or deleted among different individuals, ranging from kilobases to several megabases in length [4]. Although at least 20% of the genome was found to be copy number variable, this class of variation is, nonetheless, poorly integrated into human genetic studies. However, part of the heritability void left by GWAS could be accounted for common CNVs. Indeed, several CNVs were recently described to be associated with complex traits: a 20-kb deletion upstream of the IRGM gene with Crohn's disease [5], a 45-kb deletion upstream of NEGR1 with body mass index [6], a 32-kb deletion with psoriasis [7,8], and a 117-kb deletion of UGT2B17 with osteoporosis [9]. Consequently, the next logical step is to perform GWAS based on CNVs.

Available computer programs like Birdsuite [10], QuantiSNP [11], PennCNV [12], COKGEN [13], CNV Workshop [14] or the Affymetrix Genotyping Console allow the determination of CNVs from SNP array data. Furthermore, software systems exist for the management of genotypes, phenotypes and other subject-related information [15,16]. Unfortunately, those tools are limited either for the calculation of CNVs or the storage of phenotypes and provide no functionality to perform genome-wide association studies based on CNVs. So far, GWAS based on CNVS have used either commercial software solutions like Helixtree (Golden Helix, Inc.), Array Studio (Omicsoft), open source software like PLINK [17] or self created R scripts for the determination of genome-wide regions of interest and for the performance of statistical analysis, especially general linear regression models. The use of different software tools for each step requires additional efforts for appropriate data conversion and slows down the process as entity. For biologists without expertise in computer science or statistics these analyses turn out to be very difficult.

We present CONAN (**Co**py **N**umber Variation **An**alysis Tool), a freely available software package to support scientists by GWAS based on CNVs. It was developed with the goal of creating a user-friendly, intuitive and fast software tool which covers the whole analysis process of association studies based on CNVs. To use it in real-life scenarios, a variety of de facto standard data formats are supported (Affymetrix Genotyping Console and Microsoft Excel) and all implemented algorithms are scalable and fast enough for typical problem sizes. Moreover, visual analytical methods assist the user to get a fast overview of the results.

## Implementation

The CONAN software package consists of a client application and several database packages. The client application was implemented in Java http://www.java.com. It was successfully tested on Windows and Linux operating systems with about 1650 subjects and millions of CNVs. A user-friendly graphical interface was designed using the open source widget toolkit SWT (Standard Widget Toolkit). For wizards and progress monitor dialogs we used JFace http://wiki.eclipse.org/index.php/JFace. The complete application was programmed in a strictly object-oriented way using JFace's Action Framework and is based on the Model-View-Controller Pattern. Libraries such as JExcelApi and opencsv were used to enable the import of phenotypes and CNVs from a variety of different data formats. All needed Java libraries are included in the software package and need no additional installation.

**Figure 1:**
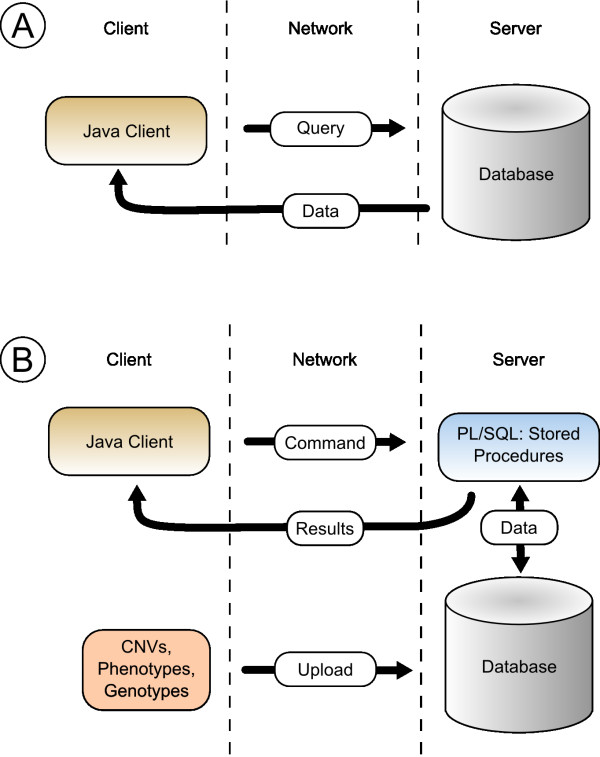
**Software Architecture**. (A) In traditional approaches the application requests data, processes it locally and uploads the results. Thus, additional amount of data transfer reduce the performance. (B) The two-tier architecture of CONAN outsources all data intensive algorithms on the database server. The client invokes stored procedures to execute them on the database server itself; thus no upload of data is required and the client retrieves only the informative results.

The users can upload their CNVs, phenotypes and genotypes directly through the client application to the server. All imported and calculated data are stored in a relational database (Oracle Database 10gR2). In order to avoid unnecessary data transfer between the client workstation and the database server, all time and data intensive analysis methods used by CONAN are executed on the database server itself. This leads to a markedly faster generation of results compared to traditional approaches where the application requests data, processes it locally and uploads the results (Figure 1). All algorithms are implemented in PL/SQL (Procedural Language/Structured Query Language) as stored procedures and are organized in several packages. The Java client uses Oracle's JDBC (Java Database Connectivity) driver to establish the connection to the database server and to invoke the stored procedures.

### CNVR detection

**Figure 2:**
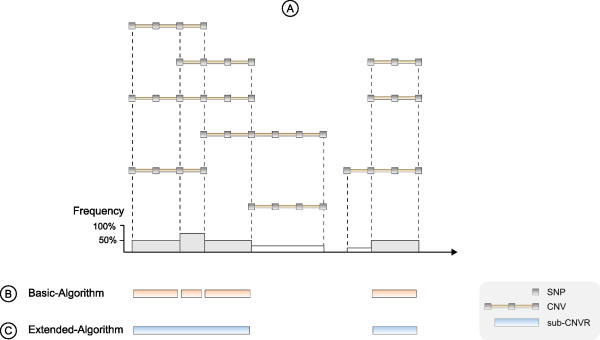
**CNVR Determination**. (A) The boundaries of a sub-CNVR are determined using the start and end SNP of each CNV. (B) The basic algorithm designates a sub-CNVR as a CNVR if its frequency is greater than the threshold. (C) The extended algorithm merges consecutive sub-CNVRs and builds a single one on their basis.

CNV regions (CNVRs) are defined as the union of all overlapping CNVs across subjects. As these regions are very long and therefore inadequate for the analysis, we divided them (based on the rules defined in [18]) into several sub-CNVRs (Figure 2A). The frequency of a sub-CNVR is defined as the percentage of subjects which have a CNV inside the boundaries. Only those with a frequency higher than the user-defined threshold are selected and saved in the database (Figure 2B).

More precisely, our CNVR algorithm performs the following steps to detect sub-CNVRs with a frequency greater than the threshold:

1. A list is created that contains only SNPs from all study individuals on a specific chromosome that define the borders of individual CNVs; upstream SNPs are designated as "starting" SNPs "S", downstream SNPs are designated as "ending" SNPs "E"

2. The list is sorted by the physical position of those SNPs (note: if several individuals have a CNV with the same starting or ending SNP, this SNP is listed for each individual separately; thus, the same SNP could be listed several times, sometimes as starting SNP, sometimes as ending SNP)

3. A counter is initiated which increments on each CNV-starting SNP and decrements on each CNV-ending SNP.

4. When two consecutive SNPs within this sorted list have different (ascending) physical positions, a next sub-CNVR could begin or previous sub-CNVR would end. The frequency of this potential sub-CNVR is determined with the help of the counter, and only if the frequency is greater than the user-specified threshold, the specific sub-CNVR is actually designated as CNVR.

5. When two consecutive SNPs within this sorted list have exactly the same physical position, the counter actualizes to the frequency of the respective CNVR as defined under step 3.

Note: the boundaries of each sub-CNVR are only approximated by the physical positions of its bracketing SNPs.

**Figure 3:**
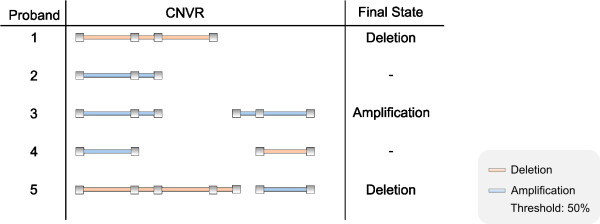
**Extended CNVR-Determination**. The longest CNV of a subject in the CNVR with a percentage greater than the threshold (e.g. 50%) is used to define the final state.

If the number of subjects is very huge and their CNVs are highly interlaced with each other, the algorithm will detect many regions with almost all of the calculated CNVRs having a length of only two SNPs. Therefore we implemented a second algorithm which extends the former one by merging consecutive sub-CNVRs with a frequency greater than the threshold and building a single one on their basis. This leads to regions with greater length, but has the consequence that the state of a subject (e.g. deletion or amplification) in a region is no longer unique. Thus we have introduced a second threshold which is used to define the state of a subject: if the CNV is the longest in the given region and its physical length is greater than this threshold, then the state of this CNV is used for the association analysis (see Figure 3).

### Association analysis

A multiple linear regression model is used to discover associations between extracted CNVRs and phenotypes. The regression analysis is performed for each CNVR separately; the dependent variable is the phenotype for which an association should be calculated. As independent variables we use the state of the subject in the region and a user defined list of covariates. Covariates are phenotypes that are used for adjustment. After the estimated coefficients and the standard variations are calculated using the Gaussian Algorithm, we determine the significance (p-value) of each region using Student's t-test. A sub-CNVR is genome-wide significant if the calculated p-value is below the Bonferroni-threshold.

## Results

CONAN is a cross-platform analysis software tool developed to provide several methods for genome-wide association studies based on copy number variations. An intuitive graphical user interface (GUI) enables the determination of CNV regions and carrying out association analysis through multiple regressions. In addition, the explorative process of results is supported by several interactive visualizations.

**Figure 4:**
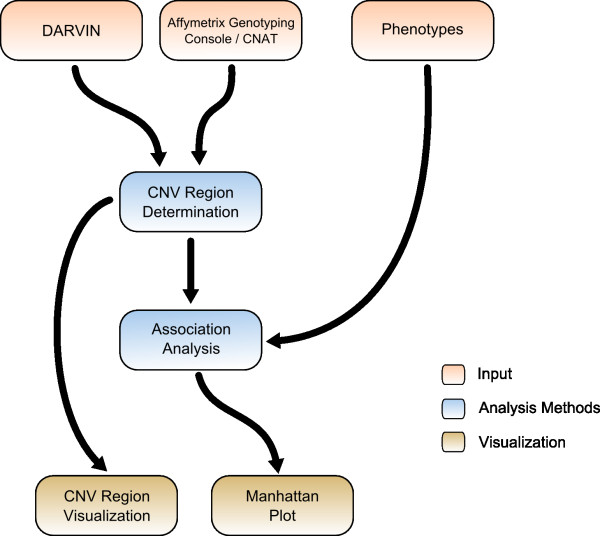
**Overview of steps**. The CONAN analysis process is divided into three main steps: data import, data analysis, and data visualization.

CONAN implements a simple but effective workflow to enable CNV analysis (Figure 4): in a first step CNVs generated by third party software are imported and stored in a relational database. In a second step, copy number variable regions (CNVR) are determined and GWA analyses are conducted. CNVRs are defined as the union of all overlapping CNVs across subjects. Finally, CONAN provides visualizations for all CNV regions and for all results of association analysis and enables thus a rapid interpretation. CONAN is very flexible and can easily be implemented in an existing workflow without error-prone data adaptation.

### User interface

**Figure 5:**
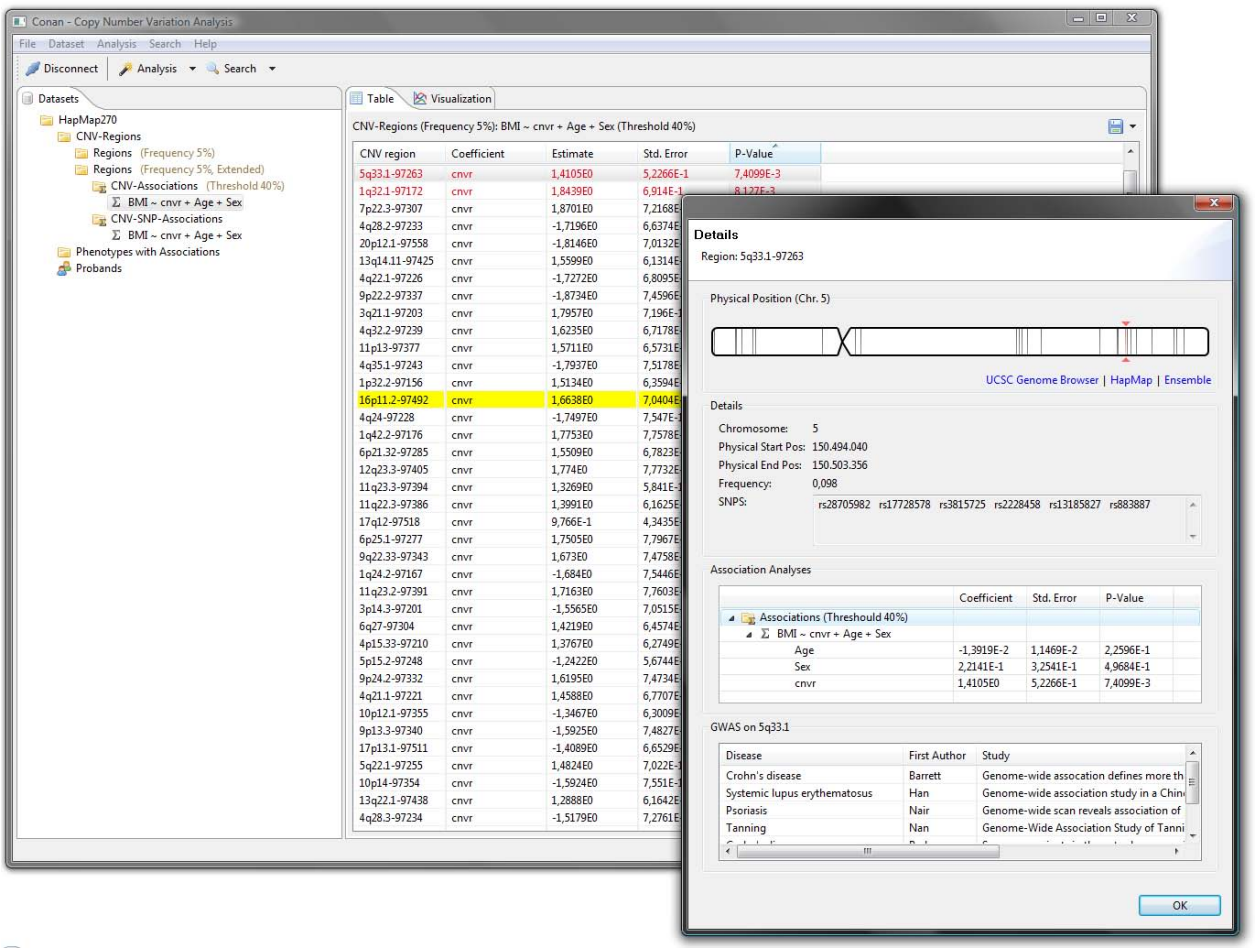
**Graphical User Interface**. All imported datasets, their calculated CNV regions and associated analyses are organized in a tree structure. All CNV regions of the current selected dataset or association analysis are shown as a table: Genome-wide significant CNVRs are highlighted in red and regions with already know associations from SNP-GWAS http://www.genome.gov/gwastudies/ are highlighted in yellow. Information about a specified CNVR is listed in a separate dialog.

CONAN has a very clear and simple interface (Figure 5): on the left side of the main window, all imported datasets, their calculated CNV regions and associated analyses are organized in a tree structure with different symbols. In the center, all CNV regions of the current selected dataset or association analysis are shown as a table (with a short summary of the parameters) and as a graphical representation. By selecting a certain CNVR a new dialog box appears which provides information about its position, its SNPs and its associations (with p-values). There is also the possibility to view the respective genomic region in the UCSC Human Genome Browser [19], HapMap Genome Browser [20] or ensemble Genome Browser [21] by just another mouse click. All algorithms and functions can be executed through well-structured menus and all required parameters can be set step by step. Moreover, the user always has the full control over the execution of each algorithm and can monitor its current progress and status.

### Data input and output

CONAN supports copy number variations which are determined using Affymetrix 500K SNP Arrays. Additionally, our solution supports the import of the "Copy Number Segment Summary" and the "Copy Number Segment Data" file format which can be exported from the frequently used Affymetrix Genotype Console software. There also exists a generic importer for CNVs that were detected from any other platform with any other software tool; however, then the CNVs need to be stored in a comma separated values file format (an example can be downloaded at http://genepi-conan.i-med.ac.at).

After the data is uploaded, a dataset is created which covers all information about the loaded CNVs and subjects. For the association analysis, different phenotypes for the same subjects are required and can be easily and at any time imported into an existing dataset. At present, CONAN allows the import of phenotypes saved in a tabular data format (Microsoft Excel or CSV) in which each row represents a certain person and contains its related phenotypic information. In addition to spreadsheet and statistic software, the efficient phenotype management software eCOMPAGT [15] can also export phenotypic data for import into CONAN. CONAN automatically checks the input files to ensure that they are corresponding to the subjects and only numerical values are contained.

For further analysis with statistical software such as R [22] and SPSS, all results can be exported as CSV (comma separated values) or Microsoft Excel files. Visualizations can be saved as high quality PNG images.

### Analysis methods

Once all data are stored in the database, the analysis process starts with the determination of CNV regions. For this purpose we have implemented the procedure described in [18,23] for the detection of regions where the number of subjects which have a CNV (with either a gain or a loss) therein is greater than a given threshold. In addition to this threshold, the user can also control the minimal number of consecutive SNPs which is used to define a CNV (CNVs, which involve less SNPs than the threshold, are discarded).

If the number of subjects is huge (>1000) and their CNVs are highly interlaced with each other, the algorithm will detect many regions with almost all of the calculated CNVRs having a length of only two SNPs. Therefore, we developed a second algorithm which extends the former procedure by merging consecutive regions and building a single one on their basis.

Table 1 summarizes several algorithm runs with different parameters to demonstrate their impact on the resulting regions. The results suggest that the number of CNV regions and the execution time depend on the total number of subjects, total number of CNVs and the threshold parameters (see "Validation" for a description of the dataset).

**Table 1:** Execution times for the calculation of CNVRs

Frequency Threshold [%]	Number of CNVRs	Execution Time [sec]
5	25,007	1,957
10	11,720	949
15	6,162	521
20	3,440	310
25	2,049	220

The analyses were run on 1,644 subjects with on average 7,130 CNVs per sample. Apart from the total number of subjects and the total number of CNVs, the resulting number of CNV regions and the execution time depend on the threshold parameters.

After CNV regions were calculated, the user is provided with the ability to perform GWA analysis on their basis. For this task we provide a multiple linear regression model (assuming an additive genetic model) which enables to discover associations between the detected regions and the imported phenotypes. A second association analysis method combines the genotyping data from SNPs with the states of detected CNVRs in order to discover associations between cumulative effect of SNPs and CNVS and phenotypes. In both cases the user can select the dependent phenotype (e.g. blood sugar level) and a list of phenotypes which should be used for adjustment (e.g. sex, age, BMI). The software automatically calculates the corresponding p-values for all selected regions and checks for genome-wide significance after Bonferroni-correction for multiple testing (p < 0.05/total number of CNV regions). In some cases it is necessary to perform the analysis only on subjects with particular properties (for example only subjects whose blood was collected after an overnight fasting period or only male subjects). Therefore, it is possible to build user defined filters in order to perform the association analysis only on a subset of all available data.

In addition, to save CPU time, already detected CNV regions can be reused for several studies. These can be compared quickly to see their difference and to identify the impact of each changed parameter.

### Data visualization

**Figure 6:**
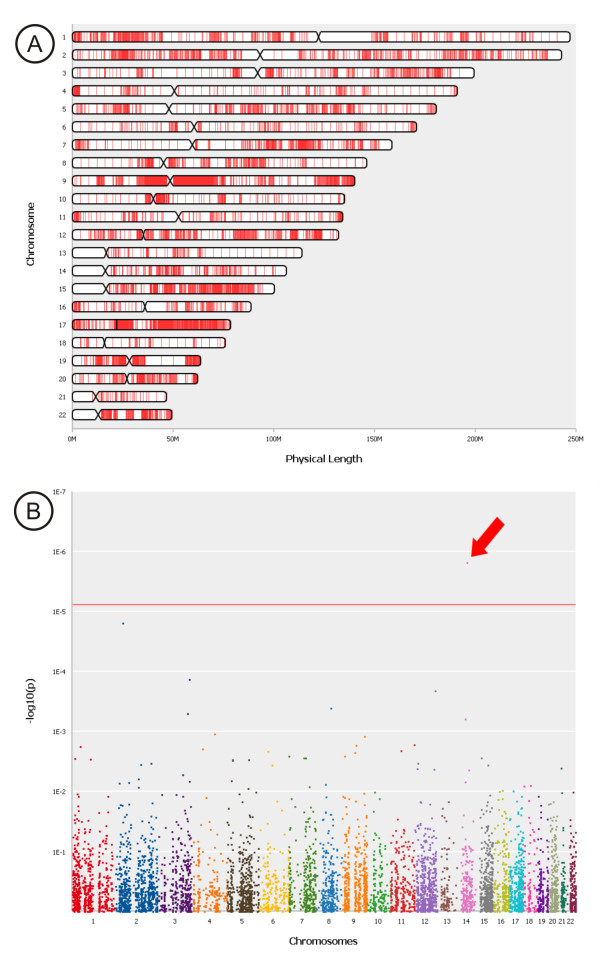
**Data Visualization**. CONAN supports the analysis process by several visualizations: (A) Visualization of the distribution of all detected CNV regions on each chromosome. (B) Visualization of associations via Manhattan plot enables a rapid identification of genome-wide significant CNVRs.

The interpretation of tables with thousands of regions is a complex and time-consuming task. Therefore, to assist the user, we have implemented several interactive visualizations to discover regions of interest in a fast manner and to show their attributes on demand. CONAN depicts the distribution of all detected CNV regions on each chromosome (Figure 6A). The results of an association study can be visualized with a Manhattan-Plot in which all p-values are plotted using log_10_-transformation and each chromosome has a different color; genome-wide significant hits can be found above the Bonferroni-threshold line which is automatically drawn considering the number of tests performed (Figure 6B). Every plotted p-value can be addressed by a mouse click, and a short overview of its properties appears. As a special feature, CONAN compares the detected regions with already known and published associations from the GWAS database [24]. Genomic regions that are known to be associated with the phenotype or disease in question are highlighted in yellow (Figure 5).

### Validation

In order to verify the implemented algorithms we have tested CONAN with sample data consisting of 1,644 KORA subjects [25]. The Affymetrix 500K SNP Chip data were analyzed by DARVIN, our in-house software solution for CNV detection using a Hidden Markov Model after identification of chromosomal gains and losses by comparing the intensity of the probe sets of all subjects with a reference set (manuscript under review). The software detected about 7,130 CNVs per sample on average. As phenotypes we used BMI, gender and age. CONAN has discovered the same associations between BMI and CNVs as previously suggested: (1) nearby gene KCTD15 [6] we have discovered a CNVR on 19q13.11 with a p-value of 0.003; (2) on 5p15.33 [18] we have discovered a CNVR with a p-value of 0.009.

## Discussion

We present CONAN, a new and useful tool for GWAS based on CNVs detected by third party computer programs. It combines the individual steps of the whole analysis process into one user-friendly software solution. Due to the outsourcing of all time intensive algorithms on the database server, the software works very fast and scales well.

The analysis of millions of CNVs is a very time-consuming task; therefore we have optimized the applied algorithms with respect to two different aspects: First we optimized the algorithm in terms of run-time and time-complexity; then we optimized the used SQL queries and created indices on the underlying tables to enable fast data retrieval. As a consequence, the import of CNVs requires more time, but as the focus of our software lies on the analysis, fast query results are more important.

### Comparison with similar software packages

The open source command line tool **Birdsuite **[10] enables the detection of CNVs and provides several scripts in order to perform GWAS on the results using PLINK [17]. Visualizations are possible with gPLINK. **SCIMMkit **[26] is also open source and provides a command line tool which enables the targeted interrogation of CNVs using Illumina Infinium II and GoldenGate SNP assays; association analysis with phenotypes is not yet provided. **Helixtree **and **Array Studio **are both commercial solutions and support a variety of input formats (CNVs detected by analyzing Affymetrix SNP arrays and Illumina arrays). GWAS are performed through a user-friendly GUI and different graphical representations enable a rapid interpretation. However, most of those approaches are client-oriented and perform their calculations locally; this leads to poor scalability and all results are stored on different workstations and not on a central machine. This is an important aspect because the amount of the genotyping data for GWAS is increasing continuously and in a non-linear manner; thus high-performance data retrieval is an important issue. CONAN solved this problem by outsourcing all tasks to a central database server and by using the client workstation only for the presentation of the results.

### Strengths and limitations

Our software has several strengths: (1) Extensive tests with real data demonstrated that the analysis of a study with about 1,600 subjects and hundreds of thousands of CNVs can be performed with CONAN without any problems and in reasonable time frames. (2) Due to an intuitive user interface and a detailed user manual, no knowledge in computer science and statistics is required to perform the association analysis. (3) With the help of the Manhattan Plot it is possible to spot within seconds which regions are genome-wide significant. In addition, various export functions enable the further usage of the newly-detected information in other software packages such as R or SPSS (see Table 2 for a complete list of all key features). CONAN has limitations as well, as it supports only phenotypes with numerical values; phenotypes at nominal level must be pre-processed and encoded numerically before they can be imported. However, the next version of CONAN is conceived to provide functions for labelling nominal variables automatically with numbers. Moreover, CONAN is presently limited to the analysis of Affymetrix SNP Arrays, but an extension to the import of Illumina data is planned for the next release. CNVs generated by QuantiSNP [11] or PennCNV [12] must be converted into a CSV file before they can be used in the software. However, a direct support of those data formats is planned. Finally, an interface between CONAN and eCOMPAGT [15,16] should eliminate the error prone export and import tasks of phenotype-data through files.

**Table 2:** Key Features

Feature	Details
**Supported CNV File Formats**	Affymetrix Genotyping Console
	Microsoft Excel
	Comma Separated Values
**Supported SNP Arrays**	Affymetrix SNP Array 500K
**Supported Phenotype File Formats**	Microsoft Excel
	Comma Separated Values
**Supported Genotype File Formats**	Affymetrix SNP 500K .call files

**Algorithms**	Genome-wide CNVR-determination
	Genome-wide CNV-phenotype association analysis

**Visualization**	Interactive Manhattan Plot with automatically drawn Bonferroni-threshold line
	Visualization of the distribution of detected CNV regions on each chromosome
	Exporting of all visualizations as PNG and JPEG images

**Analysis**	Filtering and searching of statistical results
	Highlighting of genome-wide significant results
	Highlighting of regions which fit with results from the GWAS database
	Exporting of all results as Microsoft Excel or CSV-Files
	Direct links to entries in public databases:
	UCSC Genome Browser, NCBI dbSNP, Ensembl, HapMap

## Conclusions

CONAN facilitates the performance of GWAS based on CNVs and the visual analysis of calculated results. CONAN provides a rapid, valid and straightforward software solution to identify genetic variation underlying the 'missing' heritability for complex traits that remains unexplained by recent GWAS. The freely available software can be downloaded at http://genepi-conan.i-med.ac.at.

## Availability and requirements

Project name: CONAN

Project home page: http://genepi-conan.i-med.ac.at

Operating system(s): Windows and Linux

Programming language: Java

Other requirements: Java 1.5+, relational database (Oracle)

## Authors' contributions

LF was responsible for programming and designing CONAN and drafted the manuscript. TK and FH provided CNV data from KORA. SS and HW helped with database issues. GG and HEW were responsible for the KORA study. FK and GS helped drafting the manuscript. AK-B initialized the project, supervised it and drafted the manuscript. All authors read and approved the final manuscript.
